# Supplementary material for: Modelling Skylarks (Alauda arvensis) to Predict Impacts of Changes in Land Management and Policy: Development and Testing of an Agent-Based Model
Source: PLoS One. 2013 Jun 6;8(6):e65803. doi: 10.1371/journal.pone.0065803 (PMC3675089; doi:10.1371/journal.pone.0065803)
Supplement: Supporting Information S4 — The skylark ODdox as a zipped archive. (ZIP) [file pone.0065803.s004.zip › Skylark_ODdox/globals_eval_0x66.html]

ALMaSS Skylark ODdox: File Members


ALMaSS Skylark ODdox
 2.0


- Main Page
- Related Pages
- Classes
- Files

- File List
- File Members

- All
- Functions
- Variables
- Typedefs
- Enumerations
- Enumerator
- Macros

- a
- b
- c
- d
- f
- g
- h
- i
- j
- l
- m
- p
- r
- s
- t
- w

### - f -

- fa\_ammoniumsulphate
  : treatment.h
- fa\_greenmanure
  : treatment.h
- fa\_manure
  : treatment.h
- fa\_npk
  : treatment.h
- fa\_pk
  : treatment.h
- fa\_sludge
  : treatment.h
- fa\_slurry
  : treatment.h
- flammebehandling
  : treatment.h
- fp\_greenmanure
  : treatment.h
- fp\_liquidNH3
  : treatment.h
- fp\_manganesesulphate
  : treatment.h
- fp\_manure
  : treatment.h
- fp\_npk
  : treatment.h
- fp\_npks
  : treatment.h
- fp\_pk
  : treatment.h
- fp\_sludge
  : treatment.h
- fp\_slurry
  : treatment.h
- fungicide\_treat
  : treatment.h


- Generated on Thu Jan 10 2013 13:15:39 for ALMaSS Skylark ODdox by
   1.8.1.1
